# Direct and Indirect Effects of Cytomegalovirus-Induced γδ T Cells after Kidney Transplantation

**DOI:** 10.3389/fimmu.2015.00003

**Published:** 2015-01-21

**Authors:** Lionel Couzi, Vincent Pitard, Jean-François Moreau, Pierre Merville, Julie Déchanet-Merville

**Affiliations:** ^1^Université de Bordeaux, Bordeaux, France; ^2^UMR 5164, Centre National de la Recherche Scientifique, Bordeaux, France; ^3^Service de Néphrologie, Transplantation, Dialyse, Centre Hospitalier Universitaire de Bordeaux, Bordeaux, France; ^4^Centre Hospitalier Universitaire de Bordeaux, Laboratoire d’immunologie, Bordeaux, France

**Keywords:** antibody-mediated rejection, cancer, cytomegalovirus, gamma-delta T cells, lymphocytes, renal transplantation

## Abstract

Despite effective anti-viral therapies, cytomegalovirus (CMV) is still associated with direct (CMV disease) and indirect effects (rejection and poor graft survival) in kidney transplant recipients. Recently, an unconventional T cell population (collectively designated as Vδ2^neg^ γδ T cells) has been characterized during the anti-CMV immune response in all solid-organ and bone-marrow transplant recipients, neonates, and healthy people. These CMV-induced Vδ2^neg^ γδ T cells undergo a dramatic and stable expansion after CMV infection, in a conventional “adaptive” manner. Similarly, as CMV-specific CD8+ αβ T cells, they exhibit an effector/memory TEMRA phenotype and cytotoxic effector functions. Activation of Vδ2^neg^ γδ T cells by CMV-infected cells involves the γδ T cell receptor (TCR) and still ill-defined co-stimulatory molecules such as LFA-1. A multiple of Vδ2^neg^ γδ TCR ligands are apparently recognized on CMV-infected cells, the first one identified being the major histocompatibility complex-related molecule endothelial protein C receptor. A singularity of CMV-induced Vδ2^neg^ γδ T cells is to acquire CD16 expression and to exert an antibody-dependent cell-mediated inhibition on CMV replication, which is controlled by a specific cytokine microenvironment. Beyond the well-demonstrated direct anti-CMV effect of Vδ2^neg^ γδ T cells, unexpected indirect effects of these cells have been also observed in the context of kidney transplantation. CMV-induced Vδ2^neg^ γδ T cells have been involved in surveillance of malignancy subsequent to long-term immunosuppression. Moreover, CMV-induced CD16+ γδ T cells are cell effectors of antibody-mediated rejection of kidney transplants, and represent a new physiopathological contribution to the well-known association between CMV infection and poor graft survival. All these basic and clinical studies paved the road to the development of a future γδ T cell-based immunotherapy. In the meantime, γδ T cell monitoring should prove a valuable immunological biomarker in the management of CMV infection.

## Introduction

Kidney transplantation is the treatment of choice for patients with end-stage renal failure (1, 2). However, transplantation implies long-term chronic immunosuppression to avoid acute rejection and to extend graft survival. Chronic immunosuppression reshapes host–pathogen relationships, by modifying the type or changing the magnitude of immune responses against pathogens and tumor cells. Therefore, the two main complications associated with immunosuppressive therapies are opportunistic infections and cancer.

Cytomegalovirus infection is the most frequent opportunistic infection occurring after kidney transplantation. Human cytomegalovirus (CMV) is an ubiquitous human herpesviridae, with a double-stranded linear DNA genome of 235 kb (3). Primary CMV infection in an immunocompetent host is usually asymptomatic due to the establishment of a robust and specific adaptive immune response involving CMV-specific CD4+ T cells, CD8+ T cells, and IgG, which persist lifelong. Moreover, after primo-infection, all these actors contribute to inhibit virus reactivation (3). Despite effective anti-viral therapies, CMV is still associated with CMV infection or disease in immunocompromised kidney transplant recipients (4, 5). CMV infection is characterized by CMV DNAemia (CMV DNA in blood or plasma, also called CMV viremia) regardless of symptoms and occurs in about 50% of CMV-seropositive patients (R+, patients with peripheral blood CMV IgG) (6–10), and up to 70% of donor-positive, seronegative-recipients (D+R−) in the absence of anti-viral prophylaxis (11–18). CMV disease can be a viral syndrome (CMV DNAemia with fever, malaise, leukopenia, and/or thrombocytopenia) or a tissue-invasive disease (where CMV is detected in the injured organs, mostly lungs, liver and intestines) (4, 5). It occurs in 15–20% of D+R− patients and 5–10% of R+ patients, with or without prophylaxis. Infections with high viral load require prolonged anti-viral therapy, which can lead to the emergence of CMV gene mutations associated with anti-viral resistance (mutations in *UL97* or *UL54* genes), a situation associated with high morbidity, graft loss, and death (12, 19–21). Moreover, CMV is also associated with indirect effects after kidney transplantation (22): worse patient and graft survivals (specially late-onset CMV infection or disease) (16, 23–28), more interstitial fibrosis/tubular atrophy (17), more acute rejection (17, 24, 29–31), more other opportunistic infections (32–35), an increased cardiovascular risk (36), more new-onset diabetes after transplantation (37, 38), and more graft artery stenosis (39, 40). Prophylactic anti-CMV immunoglobulin also prevents the development of early post-transplant non-Hodgkin lymphoma in kidney transplant recipients (41).

Cytomegalovirus-specific CD4+ and/or CD8+ T cell responses have been extensively documented after kidney transplantation (42–48). The efficacy of cell therapy protocols using expanded CMV-specific CD8+ T cells has demonstrated the central role played by these cells in the control of the virus (49). Therefore, it has been proposed to monitor these cells before and after transplantation to better use anti-CMV prophylaxis and therapy (50).

In 1999, we observed a massive expansion of a γδ T cell population after CMV infection in kidney transplant recipients (51, 52). This CMV-induced γδ T cell expansion did not involve the Vδ2 subset, which is usually the main subset of γδ T cells observed in the peripheral blood. Surprisingly, this increase can concern any of the Vδ1, Vδ3, and Vδ5 sub-populations (collectively designated as Vδ2^neg^ γδ T cells) (52). This initial observation, since largely confirmed by others, suggested that a population of Vδ2^neg^ γδ T cells might play an important role in the immune response to CMV infection, but raised many questions about these cells. At the afferent phase of the CMV immune response, where is their site of priming? When and how are naïve Vδ2^neg^ γδ T cells activated? At the efferent phase, where is their site of action? What is their function? When and how do they recognize target cells? This review summarizes the recent findings tentatively addressing these points and leading to the conclusion that Vδ2^neg^ γδ T cells are important actors of the anti-CMV immune response, with direct anti-CMV effects, but also unexpected indirect effects observed in the context of kidney transplantation.

## Localization of Vδ2^neg^ γδ T Cells

Once established, the expansion of circulating Vδ2^neg^ γδ T cells following CMV infection in kidney transplant recipients is prominent and stable over time (51–53). This subset, which represents 0.5% on average of the T cell pool in CMV-seronegative patients, reaches an average of 5–10% of the circulating T cell pool in CMV-seropositive patients, and up to 50% in some patients. This phenomenon is not exclusive to the kidney transplant scenario as Vδ2^neg^ γδ T cell peripheral blood expansion after CMV infection has been shown in other solid-organ transplantations (54–56), in recipients of hematopoietic stem cell transplantation (57–59), in immunodeficient children (60, 61), in neonates (62), in pregnant women (63), and in healthy individuals (64). CMV-specific CD4+ and CD8+ αβ T cells on their own already represent around 5% of the T cell pool in CMV-seropositive healthy individuals (65) and accumulate in older people (66). Vδ2^neg^ γδ T cell peripheral blood expansion further strengthens this high magnitude of the anti-CMV immune response. This accumulation of CMV-induced T cells may exert a detrimental effect on host by reducing immunity against other pathogens and could contribute to the CMV-induced immune senescence (67).

One of the most intriguing questions regarding Vδ2^neg^ γδ T cells is about their localization during the afferent and efferent phases of the immune response against CMV. To date, we still do not know where naïve Vδ2^neg^γδ T cells are primed and where they exert their function. In physiological context, Vδ2^neg^ γδ T cells are the first γδ T cell subset to emigrate from the thymus where they represent 1–15% of thymic T cells (68–71). Although poorly represented in lymph nodes, they represent 15% of T cells in the spleen where they are located in the marginal zone and red pulp (68, 69, 72). In tissues, Vδ2^neg^ γδ T cells are occasional in the kidney and the lung (68, 69). However, up to 15% of liver T cells can be γδ T cells (73–75). They are predominantly found within normal human epithelia, with a selective accumulation in intestinal and skin epithelia (76–78). In the skin, they are mainly located in the basal epithelium of epidermis, where they represent 18–29% of T cells, but they are also present in the dermis (7–9% of T cells) (69, 79–81). They express homing receptors as CCR8 and cutaneous lymphocyte-associated antigen (78, 81). The gut epithelium is where Vδ2^neg^ γδ T cells are the most abundant. They are located in the epithelium close to the basal membrane where they represent one-third of resident T cells. They are also found within the lamina propria (5% of T cells) (76, 77, 82–84). Both skin and intestinal Vδ1 repertoire are compartmentalized, with no overlap with the circulating Vδ1 repertoire, suggesting these cells are resident cells (85, 86). However, these data are counterbalanced by observations made in cattle and sheep, showing that γδ T cells could recirculate from the skin and intestinal epithelium, to the blood via afferent lymph and lymph nodes (87). Therefore in the future, the question about the localization of Vδ2^neg^ γδ T cells during the anti-CMV immune response needs to be addressed to elucidate if their peripheral blood expansion reflects an expansion from CMV-injured tissues or if blood and more probably capillaries are the theater of an immunological function of these cells. Primary CMV infection in healthy individuals initiates with replication in mucosal epithelium, a leading tissue for future Vδ2^neg^ γδ T cell exploration (3). Alternatively, endothelial cells, which are also the target of CMV express one of the Vδ2^neg^ γδ T cell receptor (TCR) ligand identified so far, endothelial protein C receptor (EPCR) (see below), and as Vδ2^neg^ γδ T cells are retrieved in vascular beds during antibody-mediated allograft rejection (see below), microcirculation should not be disregarded in these investigations.

## When do These Cells Participate to the Anti-CMV Immune Response?

The classical pathway for activating adaptive immune response and achieving a broad systemic immune response, starts with immature dendritic cells that capture pathogens and then mature and migrate to lymph nodes where they prime αβ T cells and B cells, some of which migrating back to infected tissues (88). This specific response is complemented by γδ T cells, which have the capability to recognize a large spectrum of stress-induced signals (sometimes considered as pathogen-associated-molecular patterns) and to mount local effector responses at the early stage of the immune response (89, 90). They act in synchrony with the innate immune cells as a sensor of self-dysregulation against infected or tumor cells, a function referred to as “lymphoid-stress surveillance” (89, 90). In accordance with this concept, natural and induced γδ T cell IL-17 responses occur within 12 and 60 h after stimulation, while naïve αβ T cells require antigen-specific priming and take at least 5–7 days to acquire effector function (88).

In human, early kinetics of γδ T cell response to infections are generally difficult to depict because patients present to medical care after symptom occurrence and the time of infection is not known. In this respect, post-transplantation CMV infection is a unique context because patients can be monitored before and very early after infection. In kidney transplant recipients during primo-infection, CMV-specific CD4+ T cells are detectable in the peripheral blood 7–10 days after CMV DNAemia (42, 48). CD4+ T cells are critical to control virus (44, 91). They are followed by the production of CMV IgG and CMV-specific CD8+ T cells 20 days after DNAemia (42). Surprisingly, CMV-induced Vδ2^neg^ γδ T cells undergo an expansion kinetic in the peripheral blood similar to that of CMV-specific CD8+ T cells (92). This expansion, defined as the time necessary to reach a “plateau,” although variable between patients, occurs at an average of 50 days after CMV infection (median: 45 days, min–max: 20–240 days) (93). This observation is apparently not consistent with the concept of early “lymphoid-stress surveillance.” To reconcile the late kinetics of CMV-induced Vδ2^neg^ γδ T cells with the early action of other γδ T cell populations, it has been proposed that γδ T cell populations could be divided at least in two groups: (1) innate-like cells that respond rapidly and at a relatively high frequency in many tissue sites, and (2) lymphoid-homing γδ T cells that may be primed in the circulation and clonally expanded in a conventional “adaptive” manner (90). Sampling being limited to blood of transplant recipients may have hampered detection of rapidly responding innate-like γδ T cells in CMV-infected tissues and permitted only the observation of late expanded γδ T cells in the blood. In the future, studies in animals should analyze concomitantly γδ T cells in tissues and blood, as well as their recirculation, in order to determine if a bridge exists between innate-like γδ T cells, which act at an early stage and peripheral blood CMV-induced γδ T cells, which expand later. What we can detect in blood does not necessarily represent what is going on in tissues or lymphoid organs.

## What is the Function of CMV-Induced Vδ2^neg^ γδ T Cells?

Like CD4+ T cells, there are many γδ T cell subsets with various functionalities. A large literature described their production of Th1 cytokines and their cytotoxic activity against tumor and infected cells (94–99). However, other γδ T cell sub-populations produce IL-4 and Th2 cytokines (100), are IL-17 natural or induced γδ T cells (101–103), or have characteristics of regulatory T cells (104, 105). Moreover, some γδ T cells can also regulate B cells and IgE production (100) or provide the help to rapidly generate from immature dendritic cells a pool of mature dendritic cells early during microbial invasion (106–108). Some γδ T cells can differentiate into professional antigen presenting cells, capable of inducing CD4+ T cell responses and cross-presenting soluble microbial and tumor antigens to CD8+ responder cells (109, 110). Human epidermal γδ T cells are also able to produce insulin-like growth factor 1 upon activation to control neighboring stromal cells and promote wound healing (78, 111). This high level of functional plasticity could explain why γδ T cells can be found at different locations and at different stages of the immune response.

**Figure 1:**
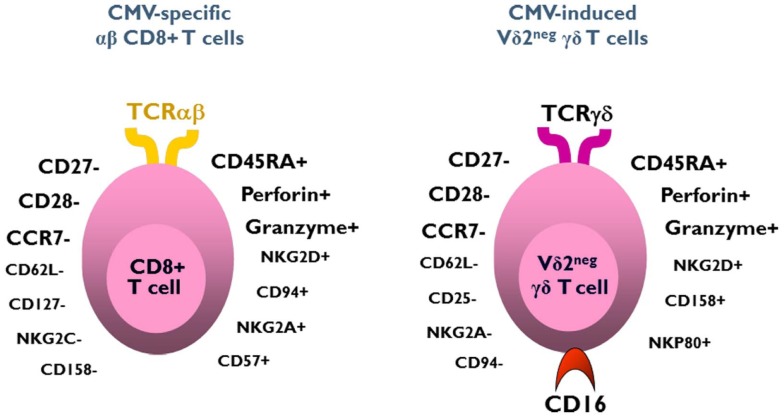
**Phenotype of CMV-induced Vδ2^neg^γδ T cells and CMV-specific CD8+ αβ T cells**. Both subsets exhibit a similar effector/memory TEMRA phenotype.

The function of CMV-induced Vδ2^neg^ γδ T cells can be first understood by analyzing their phenotype. Whereas a naive phenotype is observed in Vδ2^neg^ γδ T cells of CMV-seronegative patients, peripheral blood CMV-induced Vδ2^neg^ γδ T cells exhibit an effector/memory TEMRA phenotype, strikingly similar to and characteristic of that observed in CMV-specific CD8+ αβ T cells (112, 113). Most of these cells are CD27−, CD28−, CD45RA+, CD45RO−, Perforin ++, Granzyme B++, CCR7−, CD62L−, and have an activated phenotype (CD69+, HLA-DR+, and but CD25−), suggesting a potential cytotoxic function against CMV-infected cells (Figure 1) (52, 64, 92). A central/memory phenotype is observed less frequently than on CMV-specific CD8+ T cells (92, 112, 113). The accumulation of the TEMRA CD45RA+CD27− phenotype on both CMV-specific CD8+ T αβ cells and Vδ2^neg^ γδ T cells, suggests that this phenotype is induced by the virus (92, 114). Like the CD4+ CD28− αβ T cells and the CD8+ CD45RA+ CD27− αβ T cells described by van Lier (114), the presence of CD45RA+CD27− Vδ2^neg^ γδ T cells can also be considered as a cell signature of a “past contact with CMV” (64). The absence of these cells in the peripheral blood of patients infected with others viruses is the witness of its peculiar CMV specificity, probably under the dependence of a specific CMV-induced stress signature.

**Figure 2:**
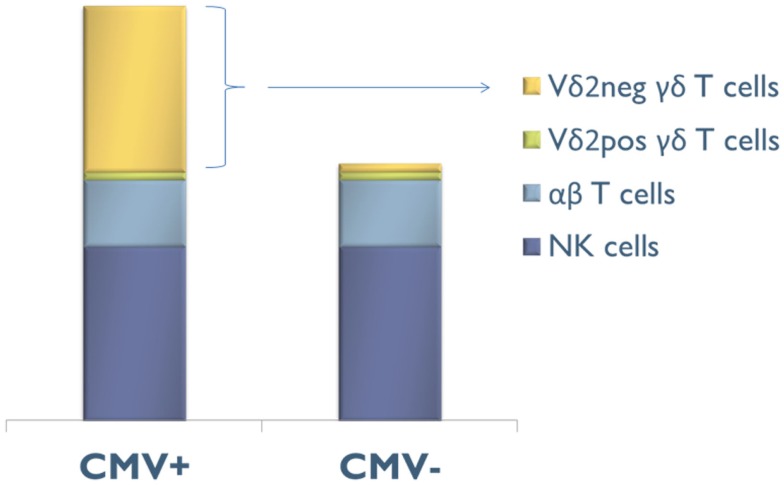
**Composition of the CD16+ lymphocyte compartment in CMV-seropositive (CMV+) and CMV-seronegative (CMV−) people**. CMV infection doubles the number of circulating CD16+ lymphocytes, through this expansion of CD16+ Vδ2^neg^γδ T cells.

Three quarters of CMV-induced Vδ2^neg^ γδ T cells also express CD16 (FcγRIIIA), which is a low-affinity receptor for Fc portion of immunoglobulin. This feature, shared with NK cells, represents a specificity of Vδ2^neg^ γδ T cells when compared to CD8 αβ T cells responding to CMV. CMV infection has therefore the unique capability to deeply reshape the CD16 compartment, because CD16 is only expressed by 20% of Vδ2^neg^ γδ T cells of CMV-seronegative patients (115). As depicted in Figure 2, CMV infection doubles the number of circulating CD16+ lymphocytes, through this expansion of CD16+ Vδ2^neg^ γδ T cells. A majority of these cells also express NK receptors (NKG2D, CD158b/j, and NKp80), by contrast to CMV-specific CD8+ T αβ cells (52, 64, 92, 115, 116). This innate-like cell phenotype probably confers to Vδ2^neg^γδ T cells a mode of activation and of regulation different from that of αβ T cells and a non-redundant role in the control of CMV. Moreover, heterogeneity in NK receptor expression can be found within a single clone of Vδ2^neg^ γδ T cells. Therefore, Vδ2^neg^ γδ T cell clones can be a mosaic of cells with similar TCR but different activating or inhibiting susceptibility, which could regulate them differently according to the context or tissues (117). In line with this singular phenotype, Vδ2^neg^ γδ T cells can be considered at the crossroads between T cells and NK cells (118, 119).

*In vitro*, Vδ2^neg^ γδ T cells are activated in the presence of free IgG-opsonized CMV or of CMV-infected fibroblast lysates, but not uninfected or other herpes virus-infected fibroblast lysates (HSV or VZV) (52). In culture with CMV-infected cells or IgG-opsonized human CMV, Vδ2^neg^ γδ T cell lines or clones coming from CMV-infected solid-organ transplant recipients produce large amounts of TNF-α and/or interferon-γ (58, 59, 62, 115, 120). *In vitro*, this CMV-induced interferon-γ production is able to inhibit CMV replication. Vδ2^neg^ γδ T cells also show perforin/granzyme B dependent cytotoxicity against CMV-infected cells *in vitro* (62, 120). All the data coming from different groups support the concept that most of the Vδ2^neg^ γδ T cells share the same cytotoxic effector function as CMV-specific CD8+ T αβ cells (42, 49). However, distinct CMV-induced Vδ2^neg^ γδ T cell clones can also provide the help to generate from immature dendritic cells a pool of mature dendritic cells (58).

In BALB/c mice and Sprague-Dawley rats, the number of γδ T cells increase after CMV infection in the draining lymph nodes, liver, peritoneal cavity, and salivary glands (121, 122). γδ T cell-depleted mice have a significantly higher viral load after CMV infection (123). Using C57BL/6 αβ and/or γδ T cell-deficient mice, we recently observed that γδ T cells were as competent as αβ T cells to control viral spread and murine CMV-induced disease and to protect mice from death (unpublished data).

All these *in vitro* indications of an anti-viral function of Vδ2^neg^γδ T cells are supported *in vivo* by the observation that early expansion of Vδ2^neg^ γδ T cells correlates with low viral loads, less symptomatic infection, and a rapid viral clearance in renal transplant patients (93).

## How do Vδ2^neg^ γδ T Cells Recognize CMV-Infected Cells or CMV?

Given their large panel of activating receptors, activation of Vδ2^neg^ γδ T cells during CMV infection may be multifactorial. We will develop here the involvement of the TCR and the CD16 molecule, which could act at different stages of the immune response. While often involved in γδ T cell activation, NKGD or its ligands (MICA/B and ULPB1-3) do not seem involved in this situation (120), probably because these γδ T cells are selected by CMV, which is able to inhibit NKG2D-ligands surface expression on infected cells (124). Two other molecules have been shown to co-stimulate activation of CMV-induced Vδ2^neg^ γδ T cells: CD8αα (58) and LFA-1, which recognizes up-regulation of ICAM-1 expression by CMV on infected cells (125).

### γδ TCR

T cell receptor involvement in Vδ2^neg^ γδ T cell reactivity against CMV-infected cells has been demonstrated by inhibition of their activation using blocking anti-TCR antibodies or through transfer of reactivity after transduction of the γδ TCR in reporter cell lines (120, 125). Analysis of γδ TCR junctional diversity shows that expansion of Vδ1 and Vδ3 T cells during CMV infection is associated with a restricted repertoire, which is suggestive of an antigens-driven selection (52, 64). This was also observed in neonates infected *in utero* with CMV, who specifically display a preponderant expansion of a particular γδ T cell population expressing a public invariant Vγ8Vδ1 TCR (62). This population has not been reported in CMV-infected adults, suggesting that it might recognize an antigen specifically induced during *in utero* infection or that this invariant TCR is generated only during fetal life. Recognition of CMV-infected cells by Vδ2^neg^ γδ T cells is independent of classical major histocompatibility complex (MHC) antigens, by contrast to CMV-specific αβ T cells. This is consistent with the reported recognition by γδ T cells of structurally diverse proteins of self and microbial origins (88), and that resembles immunoglobulin-like antigen recognition (126). Vδ1 TCR have also been shown to recognize MHC-like molecules such as MICA/B and CD1. MICA and MICB (MHC class I chain-related proteins A and B) are overexpressed in stressed cells, as in tumor or infected cells. They co-localize with Vδ1 γδ TCR in some tumors. Both γδ chains are necessary for the recognition of the MICA/B α1 and α2 domains, which is independent of any loaded peptide (94, 127–129). CD1c and CD1d are non-polymorphic molecules, which present lipids and glycolipids to NKT cells (130, 131) and also activate Vδ1 and Vδ3 γδ T cells (107, 132). Specific interaction between Vδ1 γδ TCR and CD1c molecule has been demonstrated using TCR transduction in reporter cell line, showing that no glycolipid are involved in this recognition (107). Interaction between Vδ1 γδ TCR and CD1d has also been demonstrated using tetramers, recombinants TCR, and structural studies (133–135). CD1d can be recognized by Vδ1 γδ TCR as an “unloaded” form or when loaded with endogenous glycosphingolipids (133–135) or exogenous phospholipids (108, 136).

**Figure 3:**
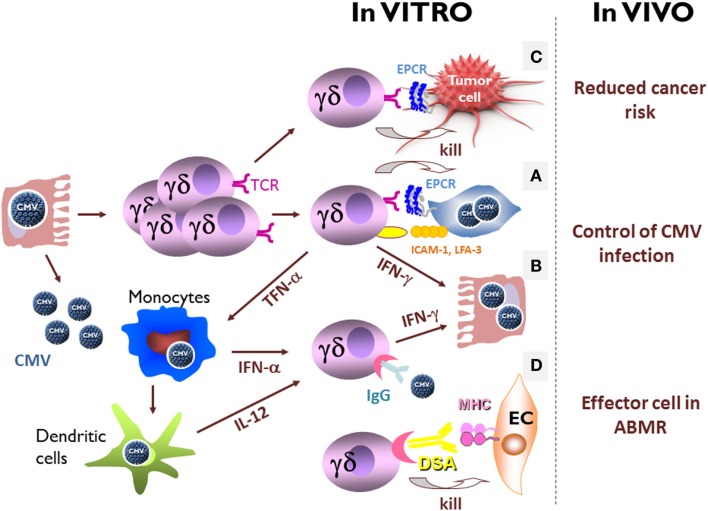
***In vitro* and *in vivo* direct and indirect effects of CMV-induced Vδ2^neg^γδ T cells**. **(A)** In culture with CMV-infected cells, Vδ2^neg^ γδ T cell lines or clones coming from CMV-infected solid-organ transplant recipients produce large amounts of TNF-α and/or interferon-γ, and exert a strong cytotoxicity against CMV-infected cells. Vδ2^neg^ γδ T cell reactivity requires EPCR expression and co-stimulatory molecules, which are over expressed in CMV-infected cells, as LFA-3 (CD2 ligand) and ICAM-1 (LFA-1 ligand). **(B)** In the absence of TCR stimulation, CD16+ Vδ2^neg^γδ T cells produce interferon-γ and inhibit CMV replication when activate by IgG-opsonized free CMV, in presence of IL-12 and interferon-α, two cytokines produced by monocytes/macrophages and dendritic cells during CMV infection. **(C)** CMV-induced Vδ2^neg^ γδ T cells have a TCR-dependent cross-reactivity against CMV-infected cells and tumor cells. **(D)** CMV-induced CD16+ Vδ2^neg^γδ T cells are able to perform antibody-dependent cell-mediated cytotoxicity (ADCC) against endothelial cells (EC) coated with donor-specific antibody (DSA). Within the grafts, γδ T cells are retrieved in close contact with endothelial cells in the peritubular capillaritis and glomerulitis associated with acute antibody-mediated rejection, only in CMV-experienced patients.

MICA/B and CD1d are not expressed on the surface of CMV-infected cells (120) and only 0.3% of CMV-induced Vδ2^neg^ γδ T cells are stained with CD1d-αGalCer tetramers (our unpublished data), suggesting that CMV does not select for MICA/B or CD1d-specific Vδ2^neg^ γδ T cells. CMV-infected cells therefore offer the opportunity to discover new Vδ2^neg^ γδ T cell ligands. Using a strategy based on the generation of monoclonal antibodies with the same antigen specificity as the CMV-induced Vδ2^neg^ γδ T cells, we identified EPCR as another MHC-like ligand for a Vγ4Vδ5 TCR (125). EPCR is a non-polymorphic protein constitutively expressed on endothelial cells and involved in the regulation of coagulation through the activation of protein C (137). It did not have any described “immunologic” function, although it displays a structural homology with CD1d (125). Recognition of EPCR by Vγ4Vδ5 TCR is independent of glycosylation and has a binding mode that does not involve discrimination of lipid antigens. Cell infection by CMV does not increase EPCR expression and Vγ4Vδ5 T cell clone reactivity requires co-stimulatory molecules, which are over expressed in CMV-infected cells, such as LFA-3 (CD2 ligand) and ICAM-1 (LFA-1 ligand) (Figure 3A) (128, 138–140). This constitutive expression of EPCR opens the possibility of its homeostatic interaction with γδ TCR, as previously reported for mice skin epithelial γδ T cells and ligands expressed on keratinocytes (141). This interaction could serve either to keep tissue γδ T cells pre-activated and ready to swiftly engage in the immune response or to activate regulatory functions necessary for maintenance of tissue integrity at steady state. Whether such a constitutively expressed TCR ligand needs conformation, topology or molecular interaction changes at the surface of target cells to prime stress surveillance response of γδ T cells deserves further investigations. Not all Vδ2^neg^ γδ T cells reactive against CMV-infected cells recognize EPCR, indicating the existence of other TCR ligands. Their characterization will be important to improve our knowledge of how cell stress and self-dysregulation are captured by Vδ2^neg^ γδ T cells.

### CD16

As mentioned above, CMV infection is associated with the expression of CD16 at the cell surface of a large majority of circulating Vδ2^neg^ γδ T cells. This expression did not allow γδ T cells to perform antibody-dependent cell-mediated cytotoxicity (ADCC) against CMV-infected cells pre-incubated with CMV hyperimmune IgGs, probably because of the seemingly low rate of IgGs directed against CMV-infected cells in sera of infected people (115). However, even in the absence of TCR stimulation, CD16+ Vδ2^neg^ γδ T cells produce interferon-γ and inhibit CMV replication when activated by IgG-opsonized free CMV, in presence of IL-12 and interferon-α, two cytokines produced by monocytes/macrophages and dendritic cells during CMV infection (Figure 3B) (115). This antibody-dependent cell-mediated inhibition (ADCI) is a new function of Vδ2^neg^ γδ T cells in their arsenal to control the virus, where antigen specificity is mediated by the antibody and not by the TCR, and is probably controlled by the cytokine microenvironment. ADCI could be restricted to specific areas, such as CMV-infected tissues or mucosa infiltrated by activated macrophages or dendritic cells, and where Vδ2^neg^ γδ T cells are homing and suspected to play a pivotal role. In accordance with the late expansion of Vδ2^neg^ γδ T in the blood during the infection, ADCI could be involved in the prevention of CMV reactivation by Vδ2^neg^ γδ T cells, when antibodies have been generated (42).

## Unexpected Anti-Tumor Effects of CMV-Induced Vδ2^neg^ γδ T Cells

Because of their immunosuppressed status, the risk of cancer in kidney transplant recipients is between 2.5 and 4 times greater than in the general population, with mainly non-melanoma skin cancer (the most common type of malignancy in kidney transplant recipients), lymphoma, cancer of the lip, vulvovaginal tumors, and kidney cancers (142–145). This is consistent with the concept of cancer immunosurveillance and cancer immunoediting, which has been well characterized in recombinase-activating gene (RAG) knock-out mice (146), as well as in humans (147–150). Among the cells involved in anti-tumor immunity, γδ T cells are considered to play a key role (95). As a major demonstration, γδ TCR knock-out mice have been shown to develop more skin cancers than wild-type mice (151). In humans, γδ T cells infiltrate many carcinomas and exert a strong interferon-γ production and cytotoxicity against carcinoma cells *in vitro* (77, 79, 81, 94–99, 151–157). More recent studies also reported opposite results suggesting pro-tumoral functions of γδ T cells both in human cancers (158) and in murine models (159–161) making the role played by the different γδ T cells in tumor surveillance more subtle. Nevertheless, during the past years, γδ T cells have been targeted in cancer immunotherapy trials showing mitigated but encouraging clinical benefit [reviewed in Ref. (162)]. It is noteworthy that all these trials uniquely targeted Vγ9Vδ2 T cells. Immunity to tumors may be acquired during events that have no clear relationship to cancer, and some infectious diseases have been associated with a reduced risk of cancers (163, 164). In line with these observations, CMV-induced Vδ2^neg^ γδ T cells have a TCR-dependent cross-reactivity against CMV-infected cells and tumor cells (Figure 3C) (58, 120). Vδ2^neg^ γδ T cell lines or clones kill tumor cells as efficiently as CMV-infected cell *in vitro*. Moreover, using a human tumor xenograft models in immunodeficient mouse, we observed that CMV-induced Vδ2^neg^ γδ T cells could inhibit tumor growth *in vivo* (165, 166). Finally in kidney transplant recipients, high CMV-induced Vδ2^neg^ γδ T cell counts as well as a past contact with CMV were associated with reduced cancer occurrence in the upcoming years (167). Taken together, these data reveal a dual role for CMV-induced Vδ2^neg^ γδ T cells in kidney transplant recipients in viral control and in surveillance of subsequent malignancy. This shared reactivity against CMV-infected and tumor cells has been observed also after allogeneic stem cell transplantation (58), where CMV infection is associated with a decreased risk of acute myeloid leukemia relapse (168, 169), and where γδ T cell expansion is associated with a reduced risk of relapse (170). This potential protective role of CMV against cancer in transplant recipients has been challenged by other groups (171), and could be in apparent contrast to the previously reported presence of the CMV genome and antigens in diverse types of carcinomas (172, 173). However, even if CMV has been suggested to play a direct role in carcinogenesis, one cannot exclude that its reactivation in tumors represents an epiphenomenon due for instance to inflammation (174, 175). All of these studies may be consistent with our results if we assume that both CMV-infected cells and tumor cells (infected or not) express the same stress-induced molecules recognized by γδ TCRs, resulting in the selection of common immune effector cells among which Vδ2^neg^ γδ T cells take an important part. They also highlight the ambiguous relationships interwoven between a virus, CMV, and its host: Parasitism or symbiosis?

## Unexpected Indirect Effect of Vδ2^neg^ γδ T during Antibody-Mediated Rejection

The epidemiological link observed between CMV and acute or chronic rejection is still not well understood. Many hypotheses have been proposed. CD4+ T cells of CMV-seropositive patients produce interferon-γ and induce both MHC class II and adhesion molecules overexpression on endothelial cells, which could potentiate *in situ* allogeneic reaction (176, 177). A cross-reactivity of CMV-specific T cells against alloantigens is also discussed (178, 179). A direct CMV effect is also likely because the persistence of the virus in the blood or the kidney leads to aggressive fibrotic lesions (26, 28, 180–182).

Recently, the importance of the recipient’s humoral response against the renal allograft has been recognized to play a key role in immunological injuries contributing to graft deterioration (183–191). Nowadays, antibody-mediated rejection is considered as the leading cause of graft loss on the long range (192). From an immunological point of view, donor-specific antibody (DSA)-mediated lesions are considered to rely on complement-fixing DSA-mediated lysis (187), direct DSA-mediated apoptosis (193), and/or ADCC by NK cells (194, 195). Until recently, complement was the most recognized way leading to graft endothelial cell injury, because deposition of C4d, a breakdown product of complement component C4, in peritubular capillaries represented the only specific tool providing the “immunopathological evidence” of DSA interaction with graft tissue (191, 196, 197). However, it does not encompass all DSA-mediated lesions (198). Glomerulitis and peritubular capillaritis, which are defined by an accumulation of polymorphonuclear cells, macrophages, and lymphocytes around capillaries, are associated with DSA, are more predictive of graft loss than C4d deposition (188, 199), and are now recognized as the main lesions of antibody-mediated rejection (200). Among these infiltrates, NK cells have recently been shown to be involved in DSA-mediated lesions of kidney microcirculation, suggesting that ADCC could play a role in DSA-mediated lesions through DSA interaction with the low-affinity Fc receptor for IgG (FcγRIIIA-CD16) expressed on NK cells (194, 195, 201). Interestingly, NK cells are not the only candidate as cell mediator of these lesions. As pointed before, CMV infection deeply reshapes the CD16+ lymphocyte compartment composition in CMV+ transplant recipients who exhibits an equal amount of CD16+ NK cells and CD16+ Vδ2^neg^ γδ T cells at the periphery (115). We have shown that CMV-induced CD16+ Vδ2^neg^ γδ T cells are able to perform ADCC against stromal cells coated with DSA *in vitro* (Figure 3D) (202). Within the grafts, γδ T cells are found in close contact with endothelial cells in the peritubular capillaritis and glomerulitis associated with acute antibody-mediated rejection, only in CMV-experienced patients. Their localization in antibody-mediated microcirculation injuries is similar to that reported for NK cells (195) and macrophages (203). Finally, an inverse correlation between a persistently increased percentage of circulating CMV-induced γδ T cells and the 1-year estimated glomerular filtration rate is observed only in kidney recipients with DSA (202). γδ T cells are usually viewed non-alloreactive because they do not recognize peptides bound to MHC molecules. However, our recent data support the conclusion that CMV-induced CD16+ γδ T cells are a new player in antibody-mediated lesions of kidney transplants. As for recognition of IgG-opsonized CMV, the antigen specificity of γδ T cell activation relies on the antibody and not on γδ TCR. Moreover, these findings suggest that γδ T cell ADCC could represent a new physiopathological contribution to the well-known but poorly understood association between CMV infection and the increased occurrence of rejection (17, 29), poor long-term graft function (16, 23, 180, 204), and low graft survival (25, 26).

In contrast to these data, two teams have proposed that Vδ1 γδ T cells play regulatory functions associated with an operational tolerance in liver transplantation (205–209). However, The Spanish team finally showed that alterations in the γδ T cell compartment were not restricted to tolerant liver recipients and confirmed the association between CMV infection and Vδ1 γδ T cell expansions (55). Most interestingly, the Japanese team described Vδ1 T cells with a public TCR infiltrating all tested tolerant liver grafts and normal livers and not found in rejected organs (209). Identification of the antigen recognized in healthy liver by this TCR could valuably contribute to decipher the mode of activation of γδ T cells with regulatory functions involved in preservation of tissue integrity.

Altogether, these data suggest that depending on the presence of CMV and/or DSA, γδ T cells could play different seemingly opposite functions on transplanted organ, which deserve further investigation in the future.

## Conclusion and Perspectives

In summary, numerous studies have now shown the involvement of Vδ2^neg^ γδ T cells within the immune response directed against CMV, with direct anti-viral effects, but also unexpected indirect effects in the context of kidney transplantation. Although most of the literature about γδ T cells considers them as actors of the innate immune response, the peripheral blood CMV-induced Vδ2^neg^ γδ T cells exhibit surprisingly at least three characteristics of the adaptive immunity. First like B cells, and αβ T cells, they use somatic rearrangement of V, D, and J genes to generate diverse antigen receptors (88). Secondly, they undergo monoclonal to polyclonal expansions, characterized by a variable extent of their repertoire from one patient to the other. Finally, these cells seem to have the ability to mount anamnestic responses, because they have the phenotype of effector/memory cells, and undergo a more rapid expansion during CMV reactivation than during primo-infection (64).

At the efferent phase of the immune response, their functions, activating pathways and kinetics have been better characterized. Understanding where, when and how naïve Vδ2^neg^ γδ T cells are activated at the afferent phase of the CMV immune response is more challenging and will most probably require *in vivo* studies in animal models. The encouraging results obtained by ours and Thomas Winkler’s team on the protective role of mouse γδ T cells against murine CMV, certainly pave the way for addressing these issues (210). Molecular understanding of how CMV-induced Vδ2^neg^ γδ T cells recognize CMV-infected cells and tumor cells necessitates the identification of representative antigenic ligands that could reveal valuable tools for vaccination trials targeting γδ T cells. An alternative is the use of γδ T cell therapy after *ex vivo* expansion of Vδ2^neg^ γδ T cells. Interesting progress has recently been made in this direction by the teams of Laurence Cooper and John Anderson who set up conditions for clinical scale propagation of polyclonal γδ T cell lines (211, 212).

All these basic and clinical studies are prerequisite to improve γδ T cell-based immunotherapy, but a shorter term use of Vδ2^neg^ γδ T cells in the clinics, will probably come from solid-organ transplantation, in which Vδ2^neg^ γδ T cell monitoring could prove a useful immunological biomarker to classify patients at risk to develop CMV infection or cancer.

Moreover, transplant patients are also prone to develop other types of infections, either parasitic (with e.g., *Toxoplasma gondii*) or bacterial (bartonella, atypical mycobacteria), which induce Vγ9Vδ2 T cell expansion due to their production of phospho-antigens. Routine monitoring of Vγ9Vδ2 T cells in our center also allowed us in several cases during the last decade to make differential diagnosis of these infections in kidney transplant recipients.

## Conflict of Interest Statement

The authors declare that the research was conducted in the absence of any commercial or financial relationships that could be construed as a potential conflict of interest.
